# Supplementary material for: An Igh distal enhancer modulates antigen receptor diversity by determining locus conformation
Source: Nat Commun. 2023 Mar 3;14:1225. doi: 10.1038/s41467-023-36414-2 (PMC9984487; doi:10.1038/s41467-023-36414-2)
Supplement: Supplementary file 1 — Supplementary Information [file 41467_2023_36414_MOESM1_ESM.pdf]

An Igh distal enhancer modulates antigen receptor diversity by determining locus conformation

Khalid H. Bhat[1,2,12], Saurabh Priyadarshi[1,12], Sarah Naiyer[1], Xinyan Qu, [1,3], Hammad Farooq[4], Eden Kleiman[5,6], Jeffery Xu, [5,7], Xue Lei, [4,8], Jose F. Cantillo[1,9] , Robert Wuerffel[1,10], Nicole Baumgarth[11], Jie Liang[4], Ann J. Feeney[5] and Amy L. Kenter[1,*]

1 Department of Microbiology and Immunology, University of Illinois College of Medicine, Chicago, IL 60612-7344, USA

2 Current address: SKUAST Kashmir, Division of Basic Science and Humanities, Faculty of Agriculture, Wadura Sopore-193201, India.

3 Current address: Medpace, Cincinnati, Ohio 45227

4 Department of Bioengineering, University of Illinois Colleges of Engineering and Medicine, Chicago, IL 60612-7344, USA

5 Department of Immunology and Microbiology, IMM-22, Scripps Research, La Jolla, CA 92037, USA

6 Current address: Crown Bioscience, San Diego, CA 92127

7 Current address: Brookwood Baptist Health General Surgery Residency. Birmingham, AL 35211

8 Current Address: Sanford Burnham Prebys Medical Discovery Institute, La Jolla, CA 92037

9 Current address: Immunotek, S.L. Alcala de Henares, Spain

10 Current address: 10441 Circle Dr. Apt 47C, Oak Lawn IL 60453

11 W. Harry Feinstone Dept. Molecular Microbiology and Immunology, Bloomberg School of Public Health, Johns Hopkins University, USA

12 Equal contributions

* Corresponding Author; e-mail: star1@uic.edu

SUPPLEMENTARY INFORMATION

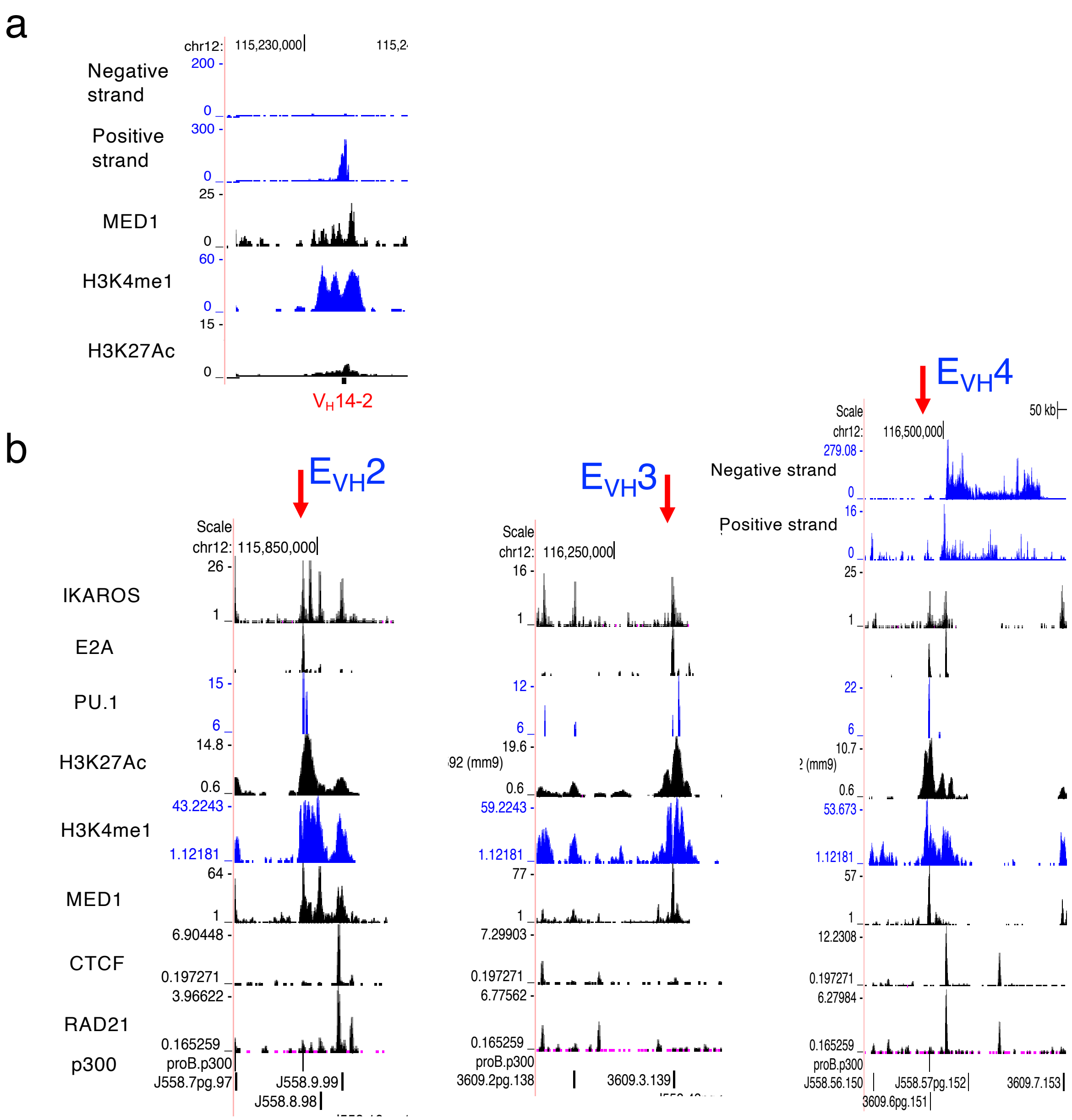

Supplementary Fig. 1. Histone protein modifications and protein factor binding factors for V_H14-2, E_{VH}2, E_{VH}3 and E_{VH}4 in the Igh locus. a) Positive and negative strand RNA-seq and ChIP-seq data for histone modifications H3K27Ac, H3K4me1, and Mediator (MED1) for the VH14-2 gene segment. **b)** ChIP-seq data for H3K27Ac, H3K4me1 and transcription factors p300, RAD21, CTCF, MED1, PU.1, E2A and IKAROS from Rag deficient pro-B cells are shown. Positive and negative strand RNA-seq is shown for E_{VH}4. The data was compiled in UCSC genome browser using mm9 genome assembly (Supplementary Table 11).

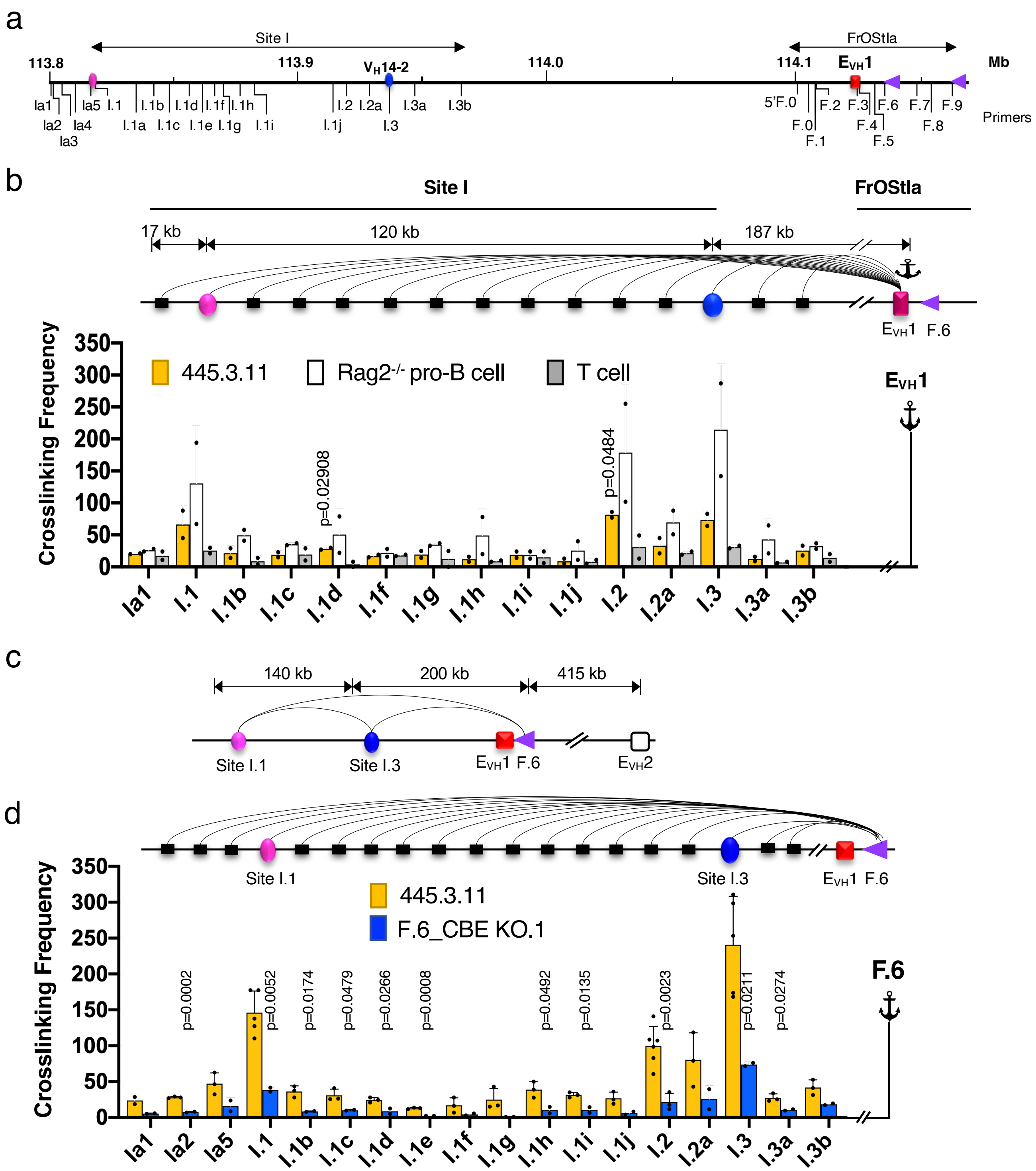

a
Site I
FrOStIa
113.8
113.9
114.0
114.1
VH14-2
EVH1
Mb
Ia1 Ia2 Ia3 Ia4 Ia5 I.1 I.1a I.1b I.1c I.1d I.1e I.1f I.1g I.1h I.1i I.1j I.2 I.2a I.3 I.3a I.3b
5'F.0 F.0 F.1 F.2 F.3 F.4 F.5 F.6 F.7 F.8 F.9
Primers
b
Site I
FrOStIa
17 kb
120 kb
187 kb
EVH1 F.6
Crosslinking Frequency
445.3.11
Rag2-/- pro-B cell
T cell
EVH1
p=0.02908
p=0.0484
Ia1 I.1 I.1b I.1c I.1d I.1f I.1g I.1h I.1i I.1j I.2 I.2a I.3 I.3a I.3b
c
140 kb
200 kb
415 kb
Site I.1
Site I.3
EVH1 F.6
EVH2
d
Site I.1
Site I.3
EVH1 F.6
Crosslinking Frequency
445.3.11
F.6_CBE KO.1
F.6
p=0.0002 p=0.0052 p=0.0174 p=0.0479 p=0.0266 p=0.0008 p=0.0492 p=0.0135 p=0.0023 p=0.0211 p=0.0274
Ia1 Ia2 Ia5 I.1 I.1b I.1c I.1d I.1e I.1f I.1g I.1h I.1i I.1j I.2 I.2a I.3 I.3a I.3b

Supplementary Fig. 2. $E_{VH}1$ and F.6 CBE anchor chromatin loops with Site I. a) Schematic of primer sites in Site I through FrOStIa highlighting DNA elements (Site I.1, pink dot; Site I.3, blue dot; $E_{VH}1$, red square; F.6 CBE, purple arrow). Primers positions are labeled and represented by the vertical bars below the line. **b,d)** Source data are provided as a Source Data file. **b)** Average crosslinking frequencies were derived from chromatin samples from Rag2-/- pro-B cells (n=2), Abl transformed 445.3.11 pro-B cell line (n=2), ConA stimulated splenic T cells (n=2). *Upper panel:* Arcs represent the 3C assays, primers are identified below the histograms, and anchor probes are indicated (anchor symbol). *Lower panels* 3C interaction profile using $E_{VH}1$ anchor probe and scanning the Site I region. P values from unpaired two-tailed Students t test. **c)** 3C interactions between anchor F.6 and 3C fragments in Site I. **d)** 3C studies using chromatin samples from the control (n=3) except for primers Ia1 (n=2), I.1 and I.3 (n=5), and I.2 (n=6) and from the F.6_CBE KO.1 (n=2) except for primers I.2 (n=3) and analyzed in duplicate. P values from unpaired two-tailed Students t test.

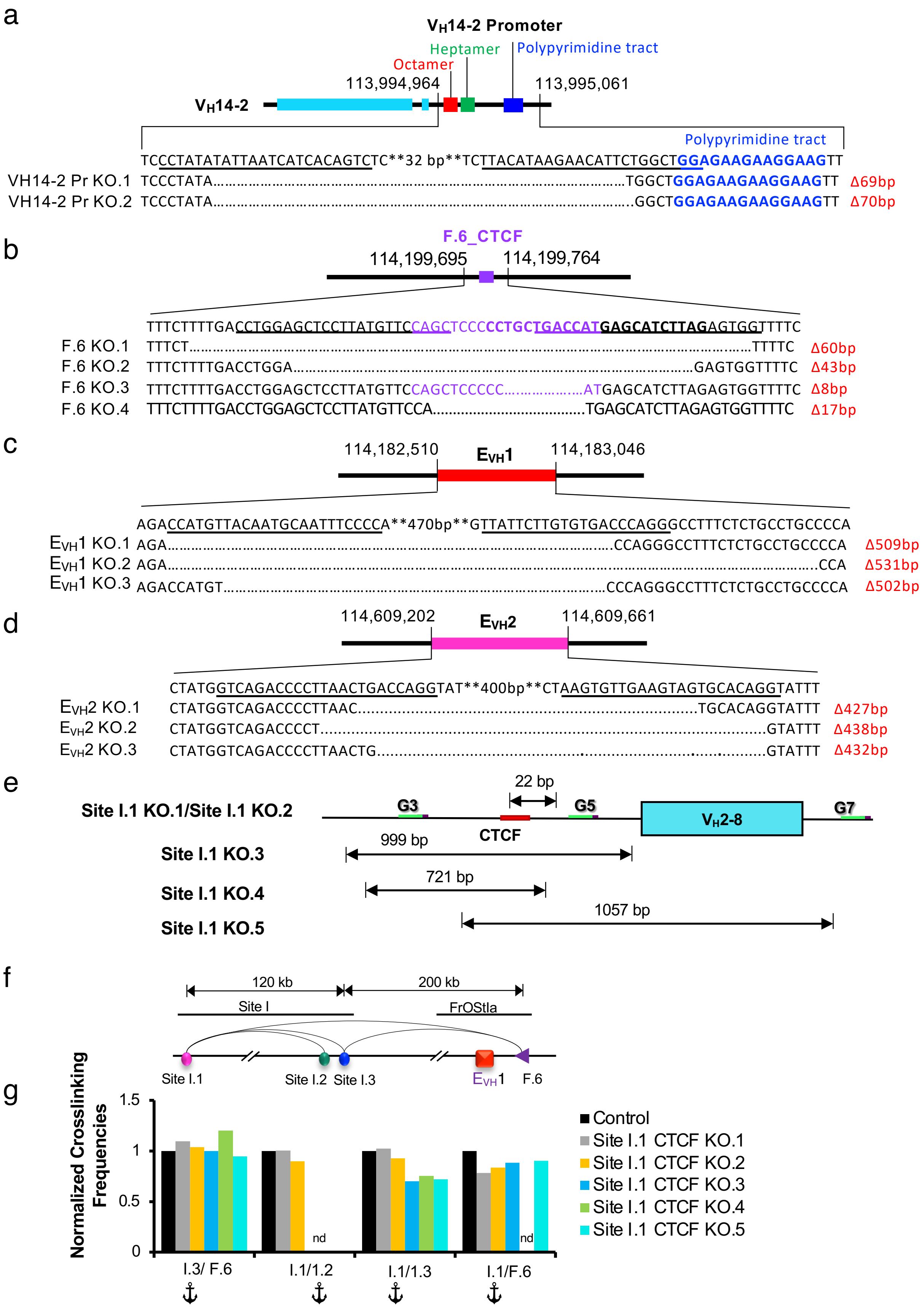

a
VH14-2 Promoter
Heptamer
Polypyrimidine tract
Octamer
113,994,964
113,995,061
VH14-2
Polypyrimidine tract
TCCCTATATATTAATCATCACAGTCTC**32 bp**TCTTACATAAGAACATTCTGGCTGGAGAAGAAGGAAGTT
VH14-2 Pr KO.1 TCCCTATA……TGGCTGGAGAAGAAGGAAGTT Δ69bp
VH14-2 Pr KO.2 TCCCTATA……GGCTGGAGAAGAAGGAAGTT Δ70bp
b
F.6_CTCF
114,199,695
114,199,764
TTTCTTTTGACCTGGAGCTCCTTATGTTCCAGCTCCCCCTGCTGACCATGAGCATCTTAGAGTGGTTTTC
F.6 KO.1 TTTCT……TTTTC Δ60bp
F.6 KO.2 TTTCTTTTGACCTGGA……GAGTGGTTTTC Δ43bp
F.6 KO.3 TTTCTTTTGACCTGGAGCTCCTTATGTTCCAGCTCCCCC……ATGAGCATCTTAGAGTGGTTTTC Δ8bp
F.6 KO.4 TTTCTTTTGACCTGGAGCTCCTTATGTTCCA……TGAGCATCTTAGAGTGGTTTTC Δ17bp
c
114,182,510
EVH1
114,183,046
AGACCATGTTACAATGCAATTTCCCCA**470bp**GTTATTCTTGTGTGACCCAGGGCCTTTCTCTGCCTGCCCCA
EVH1 KO.1 AGA……CCAGGGCCTTTCTCTGCCTGCCCCA Δ509bp
EVH1 KO.2 AGA……CCA Δ531bp
EVH1 KO.3 AGACCATGT……CCCAGGGCCTTTCTCTGCCTGCCCCA Δ502bp
d
114,609,202
EVH2
114,609,661
CTATGGTCAGACCCCTTAACTGACCAGGTAT**400bp**CTAAGTGTTGAAGTAGTGCACAGGTATTT
EVH2 KO.1 CTATGGTCAGACCCCTTAAC……TGCACAGGTATTT Δ427bp
EVH2 KO.2 CTATGGTCAGACCCCT……GTATTT Δ438bp
EVH2 KO.3 CTATGGTCAGACCCCTTAACTG……GTATTT Δ432bp
e
22 bp
Site I.1 KO.1/Site I.1 KO.2
G3
G5
VH2-8
G7
CTCF
999 bp
Site I.1 KO.3
721 bp
Site I.1 KO.4
1057 bp
Site I.1 KO.5
f
120 kb
200 kb
Site I
FrOStIa
Site I.1
Site I.2
Site I.3
EVH1
F.6
g
Normalized Crosslinking Frequencies
1.5
1
0.5
0
nd
nd
I.3/ F.6
I.1/1.2
I.1/1.3
I.1/F.6
Control
Site I.1 CTCF KO.1
Site I.1 CTCF KO.2
Site I.1 CTCF KO.3
Site I.1 CTCF KO.4
Site I.1 CTCF KO.5

Supplementary Fig. 3. CRISPR/Cas9 genome editing of TEs in a Abl-t pro-B cell line. Genomic coordinates (chr12, mm9). **a-d)** Guide (g)RNA sequences are underlined. Deletions (Δ) and their positions are displayed below the parental DNA sequence. **a**) The V_H14-2 exon and leader (cyan rectangles), octamer (red rectangle), heptamer (green rectangle), polypyrimidine tract (blue rectangle), **b**) F.6 CBE (purple rectangle). KO.1 and KO.3 were obtained using a single gRNA indicated in bold. KO.2 clone was obtained using two guides. **c**) E_{VH}1 (red rectangle). **d**) E_{VH}2 (white rectangle). **e)** Summary of the CRISPR editing at Site I.1. Guide RNAs (G3, G5, G7) used for the editing, arrows indicate the positions of deletion, numbers on the arrows indicate the base pairs deleted, red horizontal bar shows CBE, teal rectangle indicates the V_H2-8 gene. Site I.1 CTCF KO.1 and KO.2 clones were derived from parental line 445.3.11. Site I.1 CTCF KO.3, KO.4 and KO.5 clones were derived from parental line 445.3.8. **f)** Summary of the major loops tested in 3C assays (arcs) with genomic distances between them. **g)** Source data are provided as a Source Data file. Average crosslinking frequencies anchored at Site I.3 and Site I.1 from chromatin samples, 445.3.11 control (n=2); 445.3.8 control (n=2), KO lines (n=2). Average crosslinking frequencies from each control line were normalized to 1.0 (black bar), represented once by the black bar and compared with their matched KO clones (nd, not done).

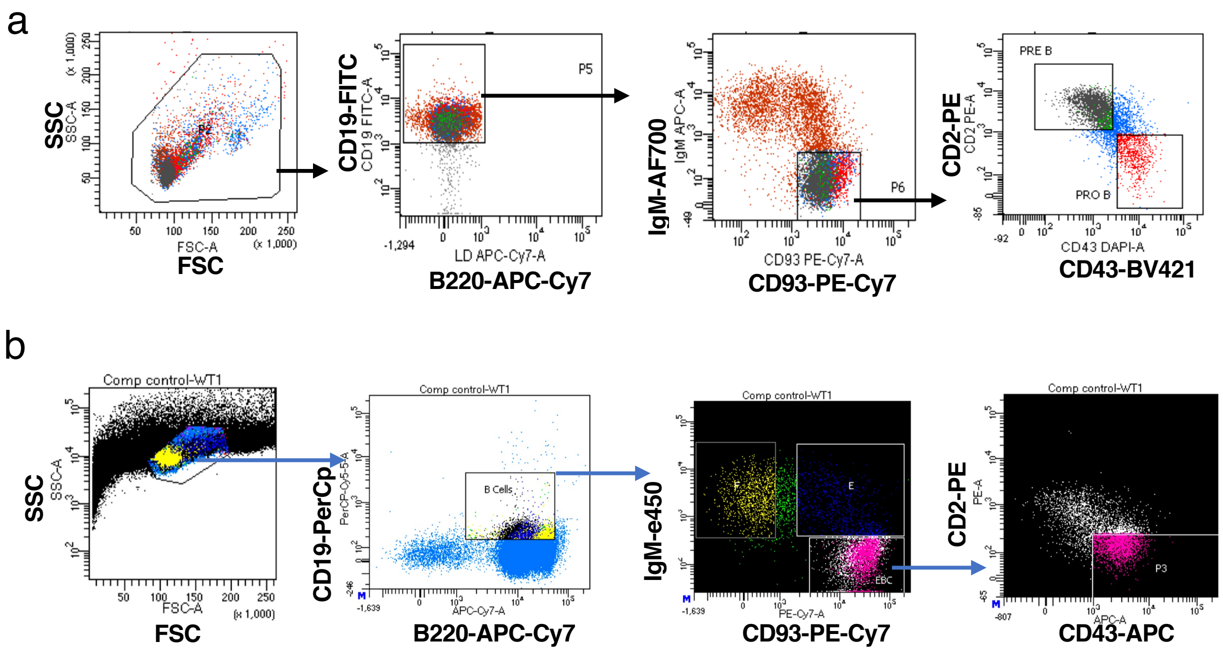

Supplementary Fig. 4. FACS gating strategy for primary pro-B cell isolation. Bone marrow (BM) was collected from humerus, tibia and femur bones of WT and $E_{VH}1^{-/-}$ mice. Representative flow cytometry results are depicted for isolation of WT pro-B cells and essentially identical results were obtained for $E_{VH}1^{-/-}$ mice. **a)** Sorting protocol for pro-B cell purification used in VDJ-seq. $CD19^+$ cells were isolated from WT and $E_{VH}1^{-/-}$ BM using CD19-conjugated MACS beads. $CD19^+$ cells were stained with antibodies to CD19-FITC, B220-APC-Cy7, CD93-PE-Cy7, CD2-PE, CD43-BV421 and IgM-AF700. Sorted pro-B cells ($CD19^+CD93^+IgM^-$ $CD2^-CD43^+$) were used to isolate gDNA. Data is representative of at least three independent experiments +/- SEM. **b)** The FACS gating strategy for pro-B cell purification used in qPCR (see Figure 4F-H). $CD19^+$ cells were first isolated using CD19-conjugated MACS beads. $CD19^+$ cells were stained with antibodies to CD19-PerCP, B220-APC-Cy7, CD93-PE-Cy7, CD2-PE, CD43-APC and IgM-e450 and were sorted on a BD FACSAria Fusion 5-18 using BD FACS Diva 8.0.1 software. FACS purified pro-B cells ($CD19^+B220^+CD93^+$ $IgM^-CD2^-CD43^+$) were used to isolate gDNA for qPCR and repertoire analysis shown in Figure 4F-H. FACS data is representative of at least three independent experiments +/- SEM.

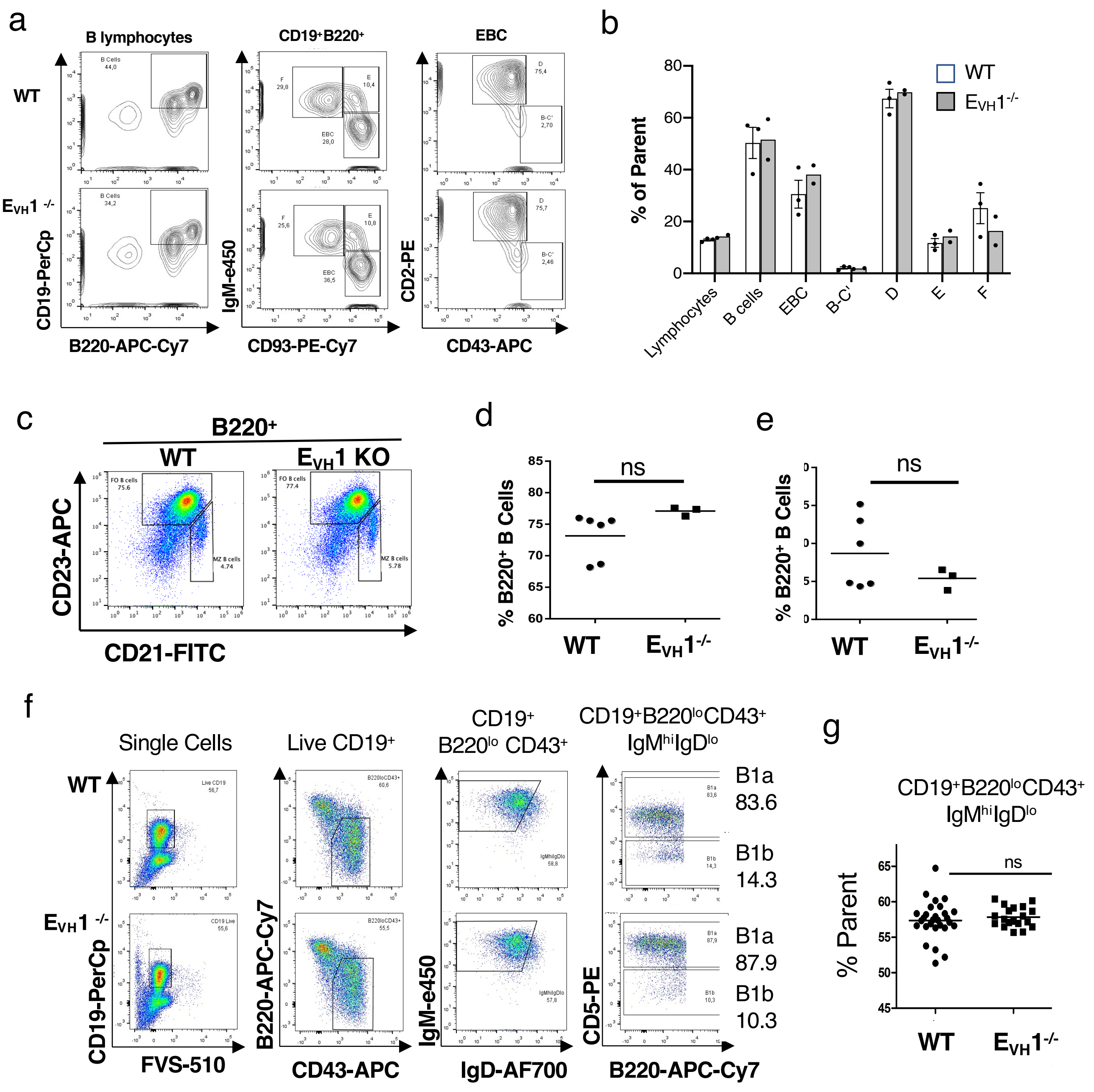

a
B lymphocytes
CD19+B220+
EBC
WT
EVH1 -/-
CD19-PerCp
B220-APC-Cy7
IgM-e450
CD93-PE-Cy7
CD2-PE
CD43-APC
b
% of Parent
WT
EVH1-/-
Lymphocytes
B cells
EBC
B-C'
D
E
F
c
B220+
WT
EVH1 KO
CD23-APC
CD21-FITC
d
ns
% B220+ B Cells
WT
EVH1-/-
e
ns
% B220+ B Cells
WT
EVH1-/-
f
Single Cells
Live CD19+
CD19+ B220lo CD43+
CD19+B220loCD43+ IgMhiIgDlo
WT
EVH1 -/-
B1a
83.6
B1b
14.3
B1a
87.9
B1b
10.3
CD19-PerCp
FVS-510
B220-APC-Cy7
CD43-APC
IgM-e450
IgD-AF700
CD5-PE
B220-APC-Cy7
g
CD19+B220loCD43+ IgMhiIgDlo
ns
% Parent
WT
EVH1-/-

Supplementary Fig. 5. B cell compartments are normal in $E_{VH}1^{-/-}$ mice. a) Analysis of Hardy fractions in BM. a) BM was collected from humerus, tibia and femur bones of WT, and $E_{VH}1^{-/-}$ mice. Cells were stained with antibodies (CD19-PerCp, B220-APC Cy7, IgM-e450, CD93-PE-Cy7, CD43-APC, CD2-PE). Representative histograms (WT, n=3; $E_{VH}1^{-/-}$, n=2) depicting different B cell fractions. B lymphocytes (B220+CD19+), early B cells (EBC; B220+CD93^{+}), pro-B cells (B220^{+}CD19^{+}CD93^{+}CD2^{-}CD43^{+}IgM$^{-;}$ similar to Hardy fractions B-C'), pre-B cells (B220^{+}CD19^{+}CD93^{+}CD2^{+}CD43^{-}IgM^{-}; similar to Hardy fraction D), immature B cells (B220^{+}CD19^{+}CD93^{+}IgM^{+}; similar to Hardy fraction E) mature B cells (B220^{+}CD19^{+}CD93^{-}IgM^{+}; similar to Hardy fraction F). **b)** The graph represents the percent of parent gated population for each early B cell fraction with SEM. (WT, n=3; $E_{VH}1^{-/-}$, n=2). **c-e)** WT, n=6; $E_{VH}1^{-/-}$, n=3 mice. **c)** Representative analyses for follicular and marginal zone B cells from C57BL/6 (WT) and $E_{VH}1^{-/-}$ spleens. Spleen cells were stained with antibodies (B220-PE, CD21-FITC and CD23-Biotin + Streptavidin-APC) and analyzed by flow cytometry for the presence of follicular (B220^{+}CD21^{lo}CD23^{hi}) and marginal zone (B220^{+} CD21^{hi}CD23^{lo}) B cells. **d,e)** Graphs for follicular **(d)** and marginal zone **(e)** B cells are a representative of two independent experiments. **e,g)** Horizontal bar indicates the mean and each symbol represents a single mouse. P values from two-tailed unpaired Student's t-test, ns = not significant. **f,g) WT,** n=35, $E_{VH}1^{-/-}$, n=19 mice. **f)** Representative analyses for peritoneal B1 B cells from WT and $E_{VH}1^{-/-}$ mice were stained with antibodies (CD19-PerCp, B220-APC Cy7, CD43 (S7)-APC, IgM-e450, IgD AF700, CD5-PE and vital dye FVS-510). **g)** The graphs represent data from three independent experiments.

Supplementary Figure 6

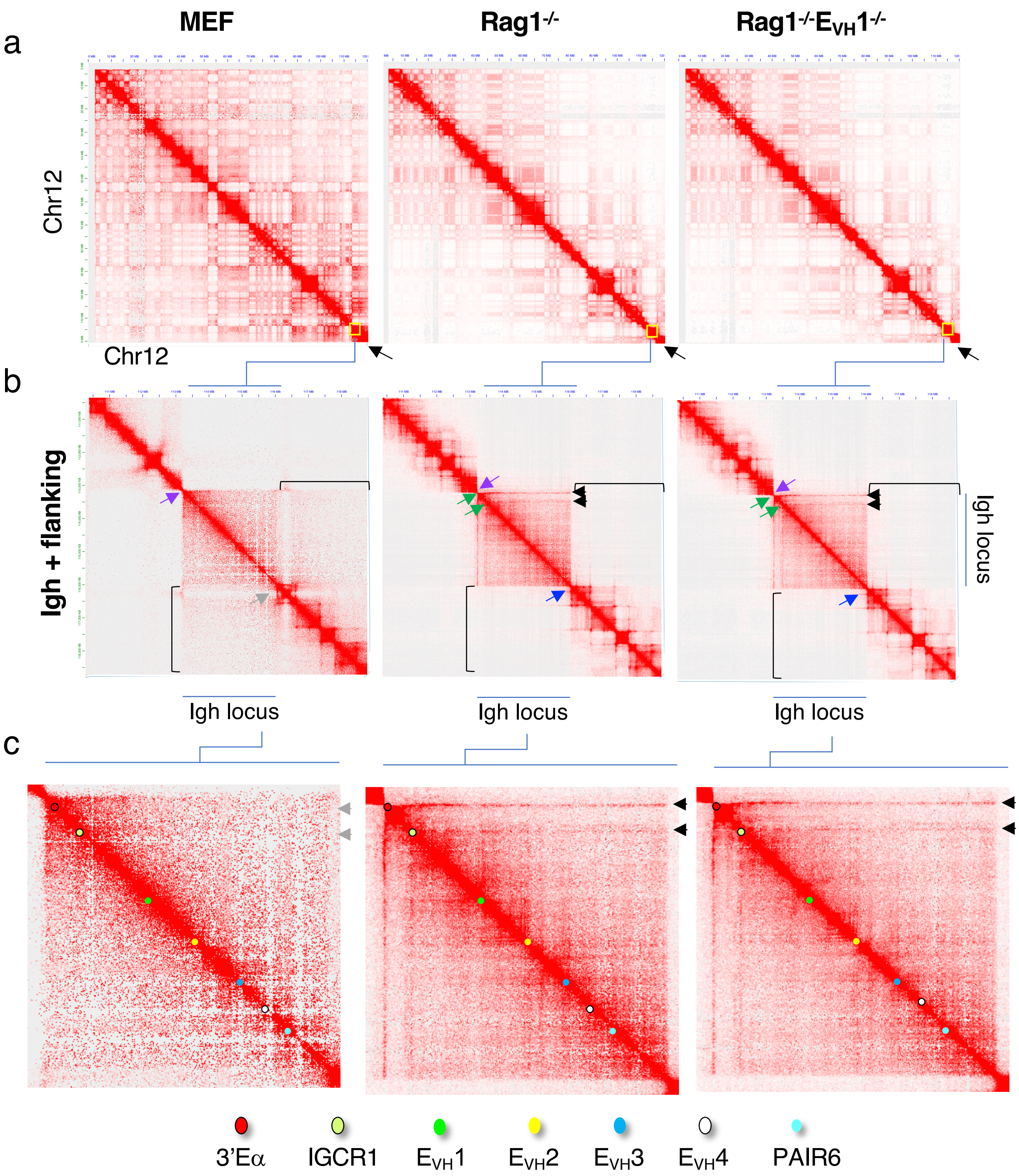

Supplementary Figure 6. **Hi-C contact maps derived from MEF, Rag1$^{-/-}$ and Rag1$^{-/-}$E$_{VH}$1 pro-B cells.** Hi-C heatmaps were generated from two biological replicates of Rag1$^{-/-}$ and Rag1$^{-/-}$E$_{VH}$1$^{-/-}$ CD19^{+} pro-B cells that yielded a minimum of 1.3 billion read pairs and 0.72 billion contacts for each sample with a 10kb bin resolution (Suppl. Table 11). The Hi-C data was KR normalized and the contact maps were generated using Juicebox and are at 10kb resolution. **a)** Hi-C heatmaps for chromosome 12 depicting compartments and TADs. Igh TAD (yellow box) indicated by the black arrow. **b)** Hi-C contact maps of the Igh locus and flanking regions spanning approximately chr12:110,500,000-118,500,000 (mm10). Igh locus TAD boundaries (3' end, purple arrow; 5' end, blue arrow; lost 5' boundary, gray arrow) Igh locus architectural stripes (black arrows). Compartments adjacent to the Igh locus (black brackets). **c)** Expanded Hi-C contact maps of the Igh locus from **(b)**. Igh locus architectural stripes are present in Rag1$^{-/-}$ and Rag1$^{-/-}$E$_{VH}$1$^{-/-}$ pro-B cells (black arrows) and absent in MEF (gray arrows). Regulatory elements (colored dots) are arrayed along the Hi-C diagonal and are identified at bottom.

Supplementary Figure 7

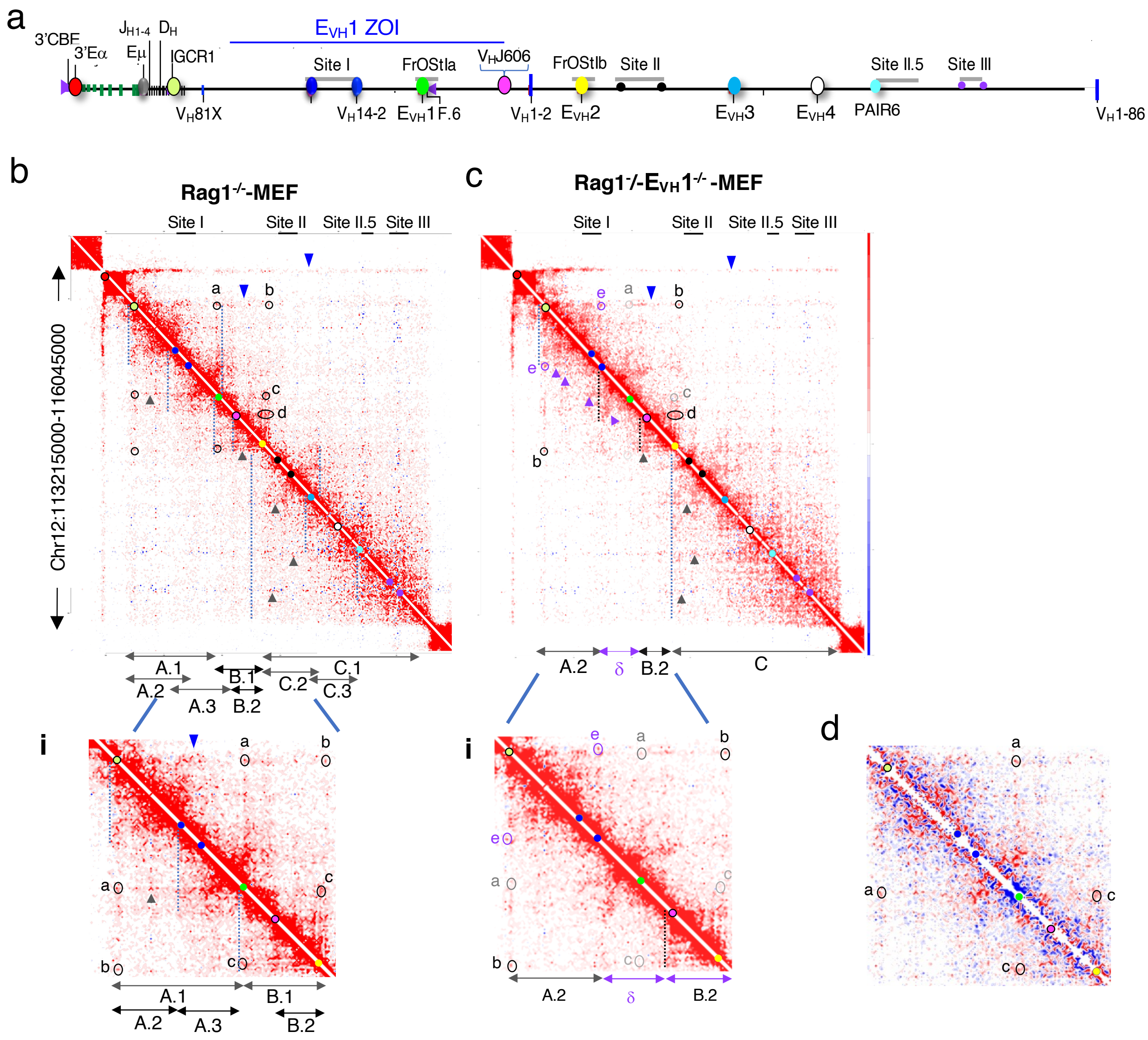

Supplementary Fig. 7. Hi-C difference maps of the Igh locus expanded for subTADs A-B. **a)** Schematic of the Igh locus with regulatory elements indicated (colored dotes) **b,c)** Quantile normalized MEF Hi-C read frequencies were subtracted from quantile normalized read frequencies of $Rag1^{-/-}$ and $Rag1^{-/-}E_{VH}1^{-/-}$ pro-B cell samples to construct difference maps. Hi-C difference maps at 10kb resolution from $Rag1^{-/-}$-MEF **(b)** and $Rag1^{-/-}E_{VH}1^{-/-}$ -MEF **(c)** pro-B cells for the Igh locus (chr12:113220000-116010000) are identical to those shown in Figure 6B. Nested loop domains (dashed vertical lines), stripes (3'->5', blue triangles; 5'->3' black triangles), dots (black circles), lost dots (gray circles). $Rag1^{-/-}E_{VH}1^{-/-}$ specific stripes (purple triangles) and dots (purple circles). Major landmarks, SiteI, SiteII, SiteII.5 and SiteIII are shown on top marked by black lines. The colored circles along the diagonal correspond to regulatory elements shown in the schematic of the Igh locus **(a)**. **bi, ci)** Expanded Hi-C difference maps spanning the region IGCR1-$E_{VH}2$ are shown for $Rag1^{-/-}$-MEF **(bi)** and $Rag1^{-/-}E_{VH}1^{-/-}$ -MEF **(Ci)** pro-B cells to highlight the differences between these genotypes. **d)** The Hi-C $Rag1^{-/-}$-MEF and $Rag1^{-/-}E_{VH}1^{-/-}$ -MEF difference maps were subtracted from each other to visualize differences between $Rag1^{-/-}$ (red intensities) versus $Rag1^{-/-}E_{VH}1^{-/-}$ (blue intensities) on the same heatmap. The expanded region spanning IGCR1-NE2 shown in the new $Rag1^{-/-}$-MEF minus $Rag1^{-/-}E_{VH}1^{-/-}$ -MEF difference map. The colored circles along the diagonal correspond to regulatory elements shown in the schematic of the Igh locus **(a)**. Selected dots (black circles) are shown as points of reference.

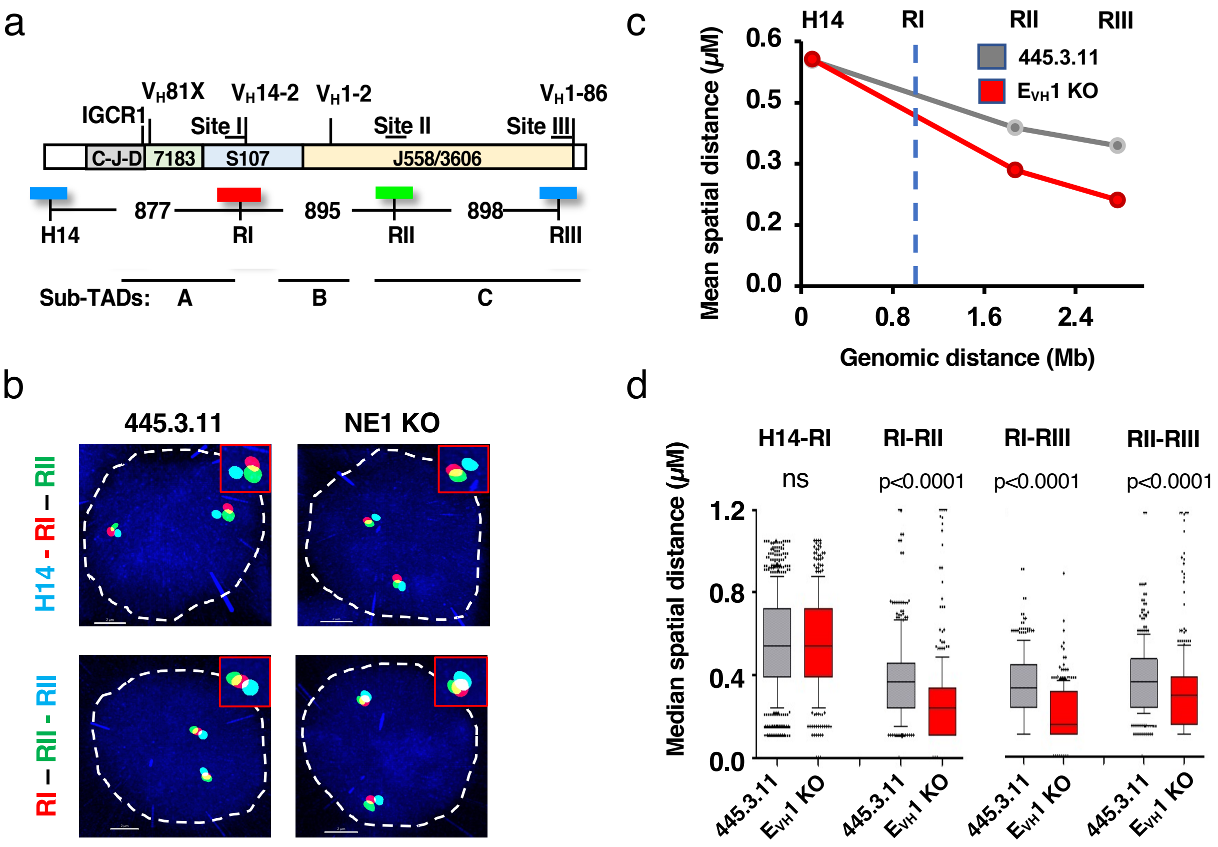

Supplementary Fig. 8. E_{VH}1 modulates Igh locus topology and compaction in Abelson transformed pro-B cell line. a) Schematic of the Igh locus. with FISH probes used in two independent experiments. BAC probes were labeled with AlexaFluor 647 (H14, RIII; blue), AlexaFluor 555 (RI; red), AlexaFluor 488 (RII; green). **b-d)** Source data are provided as a Source Data file. **b)** Representative nuclei from fixed 445.3.11 and E_{VH}1 KO cell lines hybridized simultaneously with labeled probes. N=302 alleles/genotype for the RI-RII, RI-RIII, RII-RIII probe combinations. N=922 (445.3.11 Abl-t line) and 366 (E_{VH}1 KO) for H14-RI probe combination analyzed in two independent experiments Scale = 2 μM. **c)** Mean spatial distances (y-axis) were plotted as a function of genomic distance (x-axis) for 302 alleles from two independent experiments for each probe combination. Lines indicate connectivity only. The RI anchor probe position is indicated by a vertical dashed line. **d)** Boxplots show the median (middle line), 25th and 75th percentile (box), 10th and 90th percentile (whiskers) and 10% outliers (single dots). P values (<0.0001, ****), two tail Mann-Whitney U test.

Supplementary Table 1. Antibodies and reagents

Antibodies /Reagents	Name/Clone	Source	Identifier	FACS Ab dil	Flow cytometry Ab dil	ChIP Ab dil
CD19-FITC	Rat anti mouse monoclonal/B4	Biolegend	Cat#115505 RRID:AB_313640	3/100*		
CD93 PECy7	PE/Cyanine7 anti-mouse CD93/AA4.1 (RUO)	Biolegend	Cat#136506; RRID_AB_2044012	3/100*		
IgM Fab-AF647	Alexa Fluor® 647 AffiniPure Fab Fragment Goat Anti-Mouse IgM/ Polyclonal	Jackson Immuno Research	Cat#115-607-020; RRID:AB_2338932	1.5/100*		
CD2-PE	PE anti-mouse CD2 antibody/RM2-5 (RUO)	Biolegend	Cat#100108; RRID:AB_2073690	3/100* 1/50¶	1/2500#	
CD43-BV421	BV421 Rat anti-mouse CD43/S7 (RUO)	BD Biosciences	Cat # 562958; RRID:AB_2738069	3/100*		
B220-APC-Cy7	CD45R (B220) monoclonal antibody/RA 3-6B2 (APC-eFluor 780)	eBioscience	Cat # 47-0452-82 RRID:Ab_1518810	1/100* 3/100¶	3/100#,!,$	
CD19-PerCp	PerCP anti-mouse CD19 antibody/6D5 (RUO)	Biolegend	Cat # 115532 RRID:AB_2665409	1/40¶	1/40!,$	
CD43 (S7)-APC	APC rat anti-mouse CD43/S7 (RUO)	BD Biosciences	Cat # 560663 RRID:AB_1727479	1/100¶	1/100#,!,$	
IgM-e450	IgM Monoclonal antibody/ eB121-15F9 (eFluor 450)	eBioscience ™	Cat # 48-5890-82 RRID:AB_10671539	1/50¶	1/100#,$	
CD93-PE Cy7	CD93 monoclonal antibody/AA4.1 (PE-Cyanine7)	eBioscience ™	Cat # 25-5892-81 RRID:AB_469658	1/50¶	1/50#,!	
IgD-AF700	Alexa Fluor® 700 anti-mouse IgD antibody/11-26c.2a (RUO)	Biolegend	Cat # 405730 RRID:AB_2563341		1/50$	
CD5-PE	PE anti-mouse CD5 antibody/53-7.3 (RUO)	Biolegend	Cat # 100608 RRID:AB_312737		1/1000$	
CD23-FITC	FITC anti-mouse CD23 monoclonal antibody/B3B4 (RUO)	Biolegend	Cat # 101605 RRID:AB_312830		1/50$	
CD23-biotin	Biotin Anti-Mouse CD23 /B3B4 (RUO)	BD Biosciences	Cat #553137 RRID:AB_394652		1/50!,$	
CD21-FITC	Rat Anti-CD21 / CD35 Monoclonal Antibody/7G6	BD Biosciences	Cat #553818 RRID:AB_395070		1/100!	
Streptavidin-APC	Streptavidin-allophycocyanin antibody	BD Biosciences	Cat #554067 RRID:AB_10050396		1/500!	
CD16/32 Fc Block	Purified anti-mouse CD16/32 Antibody/93 (RUO)	Biolegend	Cat# 101302; RRID:AB_312801	1/50*, ¶,#	1/50!,$	
CTCF	Rabbit Anti-CTCF Polyclonal antibody	Millipore Sigma	Cat# 07-729 RRID:AB_441965			1/450
CD19 conjugated MACS beads	CD19 microbeads mouse	Miltenyi Biotech	Cat# 130-052-201 RRID: AB_2827612			
FVS-510*	Fixable Viability Stain 510 (RUO)	BD Horizon™	Cat # 564406 RRID:AB_2869572		1/5000#,!	

* FACS: purification of pro-B cells for VDJ-seq
¶ FACS: purification of pro-B cells for qPCR repertoire analysis
# Flow cytometry analysis of pro-B cells (and Hardy fractions)
! Flow cytometry analysis of splenic B cells
$ Flow cytometry analysis of splenic B1 cells

Supplementary Table 2. Primers for qPCR and qRT-PCR assays.

Name	Sequence	Reference
Iγ2b F	ATCCCAGAGTCACAGAGGAA	1
Cγ2b R	CACACCTACAGACAACCAGAC	2
Iμ F	TCCACACAAAGACTCTGGACC	1
Cμ R	TCAGTGTTGTTCTGGTAGTTCCAG	1
V_H14-2 F	TGGAATTTCGGGGCATATTTAGTTTCACC	This study
V_H14-2 R	TCATCTTCTTCCTGATGGCAGTGGTTAC	This study
DQ52 F	CGGACAGAGCAGGCAGGTGG	3
DQ52 R	GCATCCAAGCCTCAGAAACTCAG	3
V_H81X F	AAGTGGGGGACGAGGAAGAC	3
V_H81X R	CTGGGGGGGTGTGTTTCC	3
7183 F	CACAGTGAGATTCAGAACACCCTTA	3
7183 R	GAAATGAGGAAGGCAGGCG	3
J606 F	AGGTTAGTCTGGTGAGGCATA	4
J606 R	CCAACCTACTCTAACCTCTGCTA	4
J558 F	ATTCCCCTCCCAATAGGAAA	5
J558 R	TGTCAATCACAATGGGCATC	5
VHJ558 GLT F	ATGGGATGGAGCTGGATCTT	6
VHJ558 GLT R	CTCAGGATGTGGTTACAACACTGTG	7
PAIR4 F	ATGGGGCACATAGGTTCTTCC	8
PAIR4 R	GGACATCTGAGAGATCATTGAACATC	8
VH81XRT FP	CCCAAGACATGTCATGGGAAGGGAATTC	This study
VH81XRT RP	ATGGACTTCGGGCTCAGCTTGG	This study
VHQ52.7.18 RT FP	CAGTCTGGACATGAAAGCTGCATTG	This study
VHQ52.7.18 RT RP	CTGTCCTGGTGCTGCTCC	This study
18SF	TTGACGGAAGGGCACCACCAG	9
18SR	GCACCACCACCCACGGAATCG	9

Supplementary Table 3: Guide RNAs for CRISPR/Cas9 genome editing.

Sites	gRNA	Sequence (Bold = PAM)	Clones/Mice
SiteI.3	Site I.3 g5	TTACATAAGAACATTCTGGC**TGG**	V_H14-2 Pr KO.1, V_H14-2 Pr KO.2
	Site I.3 g6	GACTGTGATGATTAATATAT**AGG**	
F.6	F.6 g1	CTAAGATGCTCATGGTCAGC**AGG**	F.6_CBE KO.1, F.6_CBE KO.3
F.6	F.6 g2	TGACCATGAGCATCTTAGAG**TGG**	F.6_CBE KO.2
	F.6 g3	GCTGGAACATAAGGAGCTCC**AGG**	
$E_{VH}1$	EVH1 g1	GGGGAAATTGCATTGTAACA**TGG**	$E_{VH}1$ KO.1, $E_{VH}1$ KO.2<br>$E_{VH}1$ KO.3
	EVH1 g3	GGGTTATTCTTGTGTGACCC**AGG**	
$E_{VH}2$	EVH2 g1	GTCAGACCCCTTAACTGACC**AGG**	$E_{VH}2$ KO.1, $E_{VH}2$ KO.2<br>$E_{VH}2$ KO.3, $E_{VH}2$ KO.4
	EVH2 g2	AAGTGTTGAAGTAGTGCAC**AGG**	
$E_{VH}1$	EVH1 g1	GGGGAAATTGCATTGTAACA**TGG**	$E_{VH}1^{-/-}$ mice
	EVH1 g3	GGGTTATTCTTGTGTGACCC**AGG**	$E_{VH}1^{-/-}$ mice

Supplementary Table 4: Primers for genotyping CRISPR/Cas9 edited cell lines and mice

Name	Sequence	Reference
c_SiteI.3 F1	AGTGAAGAGCTGGGCAAAATATAGC	This Study
c_SiteI.3 R1	GAATGCACATATTTCTGAGGCATG	This Study
c_SiteI.3 F2	ATTTGCATACTCATGAGGCAGGATC	This Study
c_SiteI.3 R2	CTGCACATGTCTCAGCGAAAT	This Study
c_F.6 F	TTCTGTCTGAGCTCCCAATTTACTTTCCTT	This Study
c_F.6 R	GGAGAAGGATTGTGAATTGTAAAATTACTCTAAATGGTT	This Study
c_EVH1 F1	GTCTAAACATATTTTGTAGGGTA	This study
c_EVH1 R1	GATCACTTACTCTCAAATCGAGAC	This study
c_EVH1 EP F	TTGTCTAAACATATTTTGTAGGGTATATTTGAA	This study
c_EVH1 EP R	GGTTCATTGAGTCACAAGGTAGTTC	This study
c_EVH1 IP F	CCATGTTACAATGCAATTTCCCC	This study
c_EVH1 IP R	CTGGGTCACACAAGAATAACCC	This study
c_EVH2 F	AAACATTTGAGGTACAGAACAT	This Study
c_EVH2 R	TCTAGGAAGACCTATTATTGCT	This Study
Rag2 WT	TCGATTCCTAGAGCGTCCTT	The Jackson Laboratory
Rag2 KO	GGTCATCCTTTGCAACACAG	The Jackson Laboratory
Rag2 Common	CAGCGCTCCTCCTGATACTC	The Jackson Laboratory
Rag1 0189	TGGATGTGGAATGTGTGCGAG	The Jackson Laboratory
Rag1 3104	CCGGACAAGTTTTTCATCGT	The Jackson Laboratory
Rag1 1746C	GAGGTTCCGCTACGACTCTG	The Jackson Laboratory

Supplementary Table 5. Primers used for D_H->J_H and V_H->D_HJ_H recombination assays.

Name	Sequence	Reference
VH81XF	GAAACTCTCCTGTGAATCCAATGAATACGAA	This study
VHJ558	CCTCCARCACAGCCTACATGSA	[11]
VH7183 F	CCGATTCACCATCTCCAGAGAC	[11]
DFL16.1 F	ACACCTGCAAAACCAGAGACCATA	[12]
JH1 R	CCCAGACATCGAAGTACCAGTAG	This study

Supplementary Table 6. Primers used for the short FISH probes

Name	Primer Sequence	Reference
Eμ F	CCCACAGGCTCGAGAACTTTAGCGAC	[13]
Eμ R	GCTGGAGAGTTAGTCCAGCCGAC	This study
Site I.3 F	GAGTAAAAAGGAATCACCCTTTTAAAACTAAGC	This study
Site I.3 R	TGAGTGTCCTTAAGTCTGCTGC	This study
E_{VH}1 F	TAGATCCCTGAAAGATGAGATTG	This study
E_{VH}1 R	CAGTTGAACCCAGAGGTAAG	This study
E_{VH}2 F	GCTCCAAGCAGAGAGTGTATTC	This study
E_{VH}2 R	CTCAGGCCCTCAAGTGTTATTC	This study
3'EαF	GGGGTAACCACGAGGAGAATATAAGC	[13]
3'EαR	GCTAGATGATGGGAGAGGATAGGTC	[13]

Supplementary Table 7. Statistical analysis of pairwise FISH probe contacts[1].

Experiments	Probes	Samples	Alleles measured[2]	P value[3]
	**Mouse**			
1	BAC H14 - RI	Rag2-/- vs Rag2-/- $E_{VH}1$-/-	(H14, RI) 400, 400	0.1702
2	BAC RI - RII - RIII	Rag2-/- vs Rag2-/- $E_{VH}1$-/-	(RI, RII) 400, 400	0.0004
			(RII, RIII) 400, 400	0.0890
			(RI, RIII) 400, 400	<0.00001
	BAC $E\mu$ - RI - RIII	Rag2-/- vs Rag2-/- $E_{VH}1$-/-	($E\mu$, RI) 922, 566	0.0350
			(R, RIII) 922, 566	<0.00001
			($E\mu$, RIII) 922, 566	<0.00001
3	$E\mu$ - $E_{VH}1$ - $E_{VH}2$	Rag2-/- vs Rag2-/- $E_{VH}1$-/-	($E\mu$, $E_{VH}1$) 400, 400	0.7231
			($E_{VH}1$, $E_{VH}2$) 400, 400	0.0312
			($E\mu$, $E_{VH}2$) 400, 400	<0.00001
	**Cell lines**			
4.1	3'Eα - $E\mu$	445.3.11 vs V_H Pr KO	(3'Eα, $E\mu$) 404, 404	0.0988
4.2		445.3.11 vs $E_{VH}1$ KO	(3'Eα, $E\mu$) 404, 404	<0.00001
4.3		445.3.11 vs F.6_CBE KO	(3'Eα, $E\mu$) 404, 404	<0.00001
5.1	$E\mu$ - Site I.3 - $E_{VH}1$	445.3.11 vs V_H Pr KO	($E\mu$, Site I.3) 404, 404	0.0015
			(Site I.3, $E_{VH}1$) 404, 404	<0.00001
			($E\mu$, $E_{VH}1$) 404, 404	0.0049
5.2	$E\mu$ - Site I.3 - $E_{VH}1$	445.3.11 vs $E_{VH}1$ KO	($E\mu$, Site I.3) 404, 404	<0.00001
			(Site I.3, $E_{VH}1$) 404, 404	<0.00001
			($E\mu$, $E_{VH}1$) 404, 404	0.0423
5.3	$E\mu$ - Site I.3 - $E_{VH}1$	445.3.11 vs F.6_CBE KO	($E\mu$, Site I.3) 404, 404	0.0088
			(Site I.3, $E_{VH}1$) 404, 404	<0.00001
			($E\mu$, $E_{VH}1$) 404, 404	<0.00001

1 Related to Figures 3, 6, 7.

2 The number of alleles for each genotype and probe pair.

3 P-values are reported comparing the pairwise spatial distance between two probes and are calculated with the Mann-Whitney U-test using GraphPad Prism software.

Supplementary Table 8: Primers for VDJ-seq.

Oligonucleotides	Sequence	Reference
**Adapter**		
VDJseq F (Mix 1)	ACACTCTTTCCCTACACGACGCTCTTCCGATCTNNNNNNGACTCG*T	[14]
VDJseq R (Mix 1)	/5Phos/CGAGTCNNNNNNAGATCGGAAGAG*C/3SpC3/	[14]
VDJseq F (Mix 2)	ACACTCTTTCCCTACACGACGCTCTTCCGATCTNNNNNNCTGCTCC*T	[14]
VDJseq R (Mix 2)	/5Phos/GGAGCAGNNNNNNAGATCGGAAGAG*C/3SpC3/	[14]
**Enrichment**	**Biotinylated extension primer sequence**	
JH1 enrichment R	/5Biosg/AGCCAGCTTACCTGAGGAGAC	This study
JH2 enrichment R	/5Biosg/GAGAGGTTGTAAGGACTCACCTG	This study
JH3 enrichment R	/5Biosg/AGTTAGGACTCACCTGCAGAGAC	This study
JH4 enrichment R	/5Biosg/AGGCCATTCTTACCTGAGGAG	This study
**PCR Primers**		
Short PE1/i5	ACACTCTTTCCCTACACGACGCTC*T	[15]
J_H1/i7 at 5' end	GTGACTGGAGTTCAGACGTGTGCTCTTCCGATCTTTACCTGAGGAGACGGTGACC*G	This study
J_H2/i7 at 5' end	GTGACTGGAGTTCAGACGTGTGCTCTTCCGATCTGGACTCACCTGAGGAGACTGTG*A	This study
J_H3/i7 at 5' end	GTGACTGGAGTTCAGACGTGTGCTCTTCCGATCTGGACTCACCTGCAGAGACAGTGA*C	This study
J_H4/i7 at 5' end	GTGACTGGAGTTCAGACGTGTGCTCTTCCGATCTCCATTCTTACCTGAGGAGACGGTG*A	This study

Supplementary Table 9. Primers and probes for 3C assays.

Primer	TagMan Probe	Sequence	Reference
Eμ		TCCACACAAAGACTCTGGACCTCT	[16]
	**A (Eμ)**	TGGCTTACCATTTGCGGTGCCTGGTTT	[16]
T.H (hs3b,4)		GCCCCTAAGACCCTACTCTGCTA	[16]
	**H (hs3b,4)**	TGACTCATCCACATCACCTTGCCT	[16]
IGCR1		AGCCTCAAGTTCCTTCAGGGC	This study
VH81X		GAACATTCTGGGAACAGTATTAGGATG	This study
Q52.2.4		CTGGATGCCTGTCTCCTGTAG	This study
Q52.3.8		CTACTGTGGTAGCTTCTACCCG	This study
7183.7.10		GTGAAGGACTTTGCAGGTTGGAC	This study
Q52.5.13		AAGACTCCCATTCTCTGAGACTGG	This study
7183.14.25		TTGCACTGCACATCACTGCTGG	This study
Ia1		GGAAGGGAATGGGCATTAATGCATTAAG	This study
Ia2		CAGCTGTAGTGATGGTAATAAAGTG	This study
Ia3		CGAGTAAATGGGGACAGGGGAAAT	This study
Ia4		CTTAGCCTTTCGGTATTCCTCC	This study
Ia5		GGTATCCAGAACTTGATGTGGTGA	This study
I.1		GTCGTCATCTACACACAGGAGC	This study
I.1a		GAAAACAAACTCAAAGACAAACTACATGATA	This study
I.1b		CCAGATACATAGAATAGGAGAGAAGAC	This study
I.1c		CGGAGAAGATCAAATTGAAAATCCTCTC	This study
I.1d		GGACCCATCCAACCATCTTTGAAATG	This study
I.1e		GACTGTTGTTGACAGATTTGGCATTC	This study
I.1f		CTGGACTGTAAAGCCAGTTCCAC	This study
I.1g		CTGGAGTCTGGCACCATCATCAT	This study
I.1h		GATAAAACTGAAGGTCTGCCCACTC	This study
I.1i		CTCTCTGAGCTCAGGAATTCCAG	This study
I.1j		CAAGAACAAATTTGAGTCAGATCTTCTG	This study
I.2		CGTTATGCTTTTAATTTGCTCACTTATAAG	This study
I.2a		CAATACCCATATGGTATTTTCAACAATTTTTG	This study
I.3		TCCTACTTCTGTCTGAGGAAATTTCG	This study
I.3a		CTCAGGAATTCGAGCAGAAGAT	This study
I.3b		ACAAACTCTGAAGAAGGTCCGAAGGT	This study
	**P_Site I.1**	CCTGTGGGACTCTAAGCCCATGTCAG	This study
	**P_Site I.3**	TGATATTGTATCTAAGTGGTCTGCACATGTC TCAG	This study
5'F.0		ATTCTTCTCAATCCTGGGACTAGT	This study
F.0		TGTCTGCATGGTGTTTCTTTCATAC	This study
F.1		TAGTGCAGCTTCCACTTAAAGAAC	This study
F.2		GACACCATGGAGATGGATCATC	This study
F.3		CCTCCTGCAGCAGGGTTATTC	This study
F.4		CCCACAAATGCCAGACTAAAGAAC	This study
F.5		CACTTTGAAGCTGAAGACATTGAC	This study
F.6		CTGAAAGCAGGCTATTCACCATG	This study
F.7		GCAAAGGACCTCTAAAGGATTGC	This study
F.8		GATTCCATAAACAGAACCCTAATGG	This study

F.9		GCCTGTTTGTTGAGAAGTGCACA	This study
	**P_F.6**	AGAGCAACAAGGAAAAGCCATCTAAGCTCCAA	This study
GD F		AGGCTTCTGACCTGCATCTTGA	[17]
GD R		TTCCAGAGCATTGTCAGCAAA	[17]
	**GD**	ACCTTGCTACTCTTCCCTGGTGTTTGTTGG	[17]
Mb1 F		CCACGCACTAGAGAGAGACTCAA	[18]
Mb1 R		CCGCCTCACTTCCTGTTCAGCCG	[18]
C14 F (hs3b,4)		ATAGGCCCTCCTCCCACCA	[16]
C14 R (hs3b,4)		CATGCGTTTGTGTCTACATGC	[16]
C3 F (Eu)		GGTATCAAAGGACAGTGCTTAG	This study
C3 R (Eu)		CAGCCTAGTTTAGCTTAGCG	This study

Supplementary Table 10. In situ Hi-C Library Statistics

	Rag1-/-1	Rag1-/-2	Rag1-/-_merged_replicates
Sequenced Read Pairs	704,893,796	595,148,192	1,300,041,988
Normal Paired	145,215,091 (20.60%)	147,945,053 (24.86%)	293,160,144 (22.55%)
Chimeric Paired	475,045,221 (67.39%)	376,176,420 (63.21%)	851,221,641 (65.48%)
Chimeric Ambiguous	81,395,262 (11.55%)	68,035,698 (11.43%)	149,430,960 (11.49%)
Unmapped	3,238,222 (0.46%)	2,991,021 (0.50%)	6,229,243 (0.48%)
Alignable (Normal+Chimeric Paired)	620,260,312 (87.99%)	524,121,473 (88.07%)	1,144,381,785 (88.03%)
Unique Reads	478,530,695 (67.89%)	414,774,186 (69.69%)	885,121,908 (68.08%)
PCR Duplicates	136,259,276 (19.33%)	104,319,997 (17.53%)	248,853,403 (19.14%)
Optical Duplicates	5,470,341 (0.78%)	5,027,290 (0.84%)	10,406,474 (0.80%)
Library Complexity Estimate	1,173,386,201	1,111,955,039	2,189,931,487
Below MAPQ Threshold	85,367,316 (12.11% / 17.84%)	75,979,254 (12.77% / 18.32%)	160,175,973 (12.32% / 18.10%)
Hi-C Contacts	393,163,379 (55.78% / 82.16%)	338,794,932 (56.93% / 81.68%)	724,945,935 (55.76% / 81.90%)
Inter-chromosomal	60,362,190 (8.56% / 12.61%)	53,686,252 (9.02% / 12.94%)	114,046,736 (8.77% / 12.88%)
Intra-chromosomal	332,801,189 (47.21% / 69.55%)	285,108,680 (47.91% / 68.74%)	610,899,199 (46.99% / 69.02%)
Short Range (<20Kb)	124,066,290 (17.60% / 25.93%)	99,751,406 (16.76% / 24.05%)	216,820,141 (16.68% / 24.50%)
Long Range (>20Kb)	208,725,177 (29.61% / 43.62%)	185,347,443 (31.14% / 44.69%)	394,059,576 (30.31% / 44.52%)

	Rag1-/-NE1-/-1	Rag1-/-NE1-/-2	Rag1-/-NE1-/-_merged_replicates
Sequenced Read Pairs	761,741,401	736,789,530	1,498,530,931
Normal Paired	170,981,180 (22.45%)	161,579,433 (21.93%)	332,560,613 (22.19%)
Chimeric Paired	501,909,501 (65.89%)	490,577,891 (66.58%)	992,487,392 (66.23%)
Chimeric Ambiguous	85,210,925 (11.19%)	81,411,068 (11.05%)	166,621,993 (11.12%)
Unmapped	3,639,795 (0.48%)	3,221,138 (0.44%)	6,860,933 (0.46%)
Alignable (Normal+Chimeric Paired)	672,890,681 (88.34%)	652,157,324 (88.51%)	1,325,048,005 (88.42%)
Unique Reads	517,825,831 (67.98%)	507,477,961 (68.88%)	1,014,856,941 (67.72%)
PCR Duplicates	148,381,644 (19.48%)	138,830,218 (18.84%)	297,781,998 (19.87%)
Optical Duplicates	6,683,206 (0.88%)	5,849,145 (0.79%)	12,409,066 (0.83%)
Library Complexity Estimate	1,264,111,007	1,280,223,735	2,436,635,481
Below MAPQ Threshold	92,053,634 (12.08% / 17.78%)	89,227,706 (12.11% / 17.58%)	179,928,939 (12.01% / 17.73%)
Hi-C Contacts	425,772,197 (55.89% / 82.22%)	418,250,255 (56.77% / 82.42%)	834,928,002 (55.72% / 82.27%)
Inter-chromosomal	42,925,248 (5.64% / 8.29%)	42,718,155 (5.80% / 8.42%)	85,641,452 (5.72% / 8.44%)
Intra-chromosomal	382,846,949 (50.26% / 73.93%)	375,532,100 (50.97% / 74.00%)	749,286,550 (50.00% / 73.83%)
Short Range (<20Kb)	137,296,147 (18.02% / 26.51%)	135,640,527 (18.41% / 26.73%)	263,861,777 (17.61% / 26.00%)
Long Range (>20Kb)	245,542,117 (32.23% / 47.42%)	239,883,125 (32.56% / 47.27%)	485,407,716 (32.39% / 47.83%)

Supplementary Table 11. Public ChIP-seq data sets from Rag deficient pro-B cells.

ChIP Seq1 *	Build	GEO accession #	File type	Reference
Brg1	Mm9	GSM1635413	Wig	19
Ikaros	Mm9	GSM1635411	Bedgraph	19
E2A	Mm8	GSM546523	Bed	20
Med1	Mm9	GSM1038263	Wig	21
H3K27ac	Mm9	GSM2255552	BigWig	22
H3K4me1	Mm8	GSM546527	BigWig	20
PU.1	Mm9	GSM539537	Wig	23
P300	Mm9	GSM987808	Bed	24
H3K4me3	Mm9	GSM4350110	BigWig	25
Pax5	Mm9	GSM4805384	Bedgraph	26
IRF4	Mm9	GSM1296534	BigWig	27
CTCF	Mm9	GSM1156665	BigWig	28
Rad21	Mm9	GSM1156667	BigWig	28
RNA_Seq	Mm9	GSM1006370	Bedgraph	29

* All data sets were converted to mm9.

REFERENCES

1 Shen, H. M. *et al.* Loop extrusion promotes an alternate pathway for isotype switching. *Cell Rep* **37**, 110059, doi:10.1016/j.celrep.2021.110059 (2021).

2 Dai, H. Q. *et al.* Loop extrusion mediates physiological Igh locus contraction for RAG scanning. *Nature* **590**, 338-343, doi:10.1038/s41586-020-03121-7 (2021).

3 Oudinet, C., Braikia, F. Z., Dauba, A. & Khamlichi, A. A. Recombination may occur in the absence of transcription in the immunoglobulin heavy chain recombination centre. *Nucleic Acids Res*, doi:10.1093/nar/gkaa108 (2020).

4 Braikia, F. Z. *et al.* Developmental Switch in the Transcriptional Activity of a Long-Range Regulatory Element. *Mol Cell Biol* **35**, 3370-3380, doi:10.1128/MCB.00509-15 (2015).

5 Bolland, D. J. *et al.* Antisense intergenic transcription in V(D)J recombination. *Nat Immunol* **5**, 630-637, doi:10.1038/ni1068 (2004).

6 Guo, C. *et al.* CTCF-binding elements mediate control of V(D)J recombination. *Nature* **477**, 424-430, doi:10.1038/nature10495 (2011).

7 Perlot, T., Alt, F. W., Bassing, C. H., Suh, H. & Pinaud, E. Elucidation of IgH intronic enhancer functions via germ-line deletion. *Proc Natl Acad Sci U S A* **102**, 14362-14367, doi:10.1073/pnas.0507090102 (2005).

8 Puget, N. *et al.* Insertion of an imprinted insulator into the IgH locus reveals developmentally regulated, transcription-dependent control of V(D)J recombination. *Mol Cell Biol* **35**, 529-543, doi:10.1128/MCB.00235-14 (2015).

9 Rhinn, H. *et al.* Housekeeping while brain's storming Validation of normalizing factors for gene expression studies in a murine model of traumatic brain injury. *BMC Mol Biol* **9**, 62, doi:1471-2199-9-62 [pii] 10.1186/1471-2199-9-62 (2008).

10 Espinoza, C. R. & Feeney, A. J. The extent of histone acetylation correlates with the differential rearrangement frequency of individual VH genes in pro-B cells. *J Immunol* **175**, 6668-6675, doi:10.4049/jimmunol.175.10.6668 (2005).

11 Volpi, S. A. *et al.* Germline deletion of Igh 3' regulatory region elements hs 5, 6, 7 (hs5-7) affects B cell-specific regulation, rearrangement, and insulation of the Igh locus. *J Immunol* **188**, 2556-2566, doi:10.4049/jimmunol.1102763 (2012).

12 Subrahmanyam, R. *et al.* Localized epigenetic changes induced by DH recombination restricts recombinase to DJH junctions. *Nature immunology* **13**, 1205-1212, doi:10.1038/ni.2447 (2012).

13 Gerasimova, T. *et al.* A structural hierarchy mediated by multiple nuclear factors establishes IgH locus conformation. *Genes Dev* **29**, 1683-1695, doi:10.1101/gad.263871.115 (2015).

14 Matheson, L. S. *et al.* Local Chromatin Features Including PU.1 and IKAROS Binding and H3K4 Methylation Shape the Repertoire of Immunoglobulin Kappa Genes Chosen for V(D)J Recombination. *Front Immunol* **8**, 1550, doi:10.3389/fimmu.2017.01550 (2017).

15 Kleiman, E., Loguercio, S. & Feeney, A. J. Epigenetic Enhancer Marks and Transcription Factor Binding Influence Vkappa Gene Rearrangement in Pre-B Cells and Pro-B Cells. *Front Immunol* **9**, 2074, doi:10.3389/fimmu.2018.02074 (2018).

16 Feldman, S. *et al.* Constraints contributed by chromatin looping limit recombination targeting during Ig class switch recombination. *J Immunol* **194**, 2380-2389, doi:10.4049/jimmunol.1401170 (2015).

17 Kumar, S. *et al.* Flexible ordering of antibody class switch and V(D)J joining during B-cell ontogeny. *Genes Dev* **27**, 2439-2444, doi:10.1101/gad.227165.113 (2013).

18 Wang, L., Whang, N., Wuerffel, R. & Kenter, A. L. AID-dependent histone acetylation is detected in immunoglobulin S regions. *J Exp Med* **203**, 215-226, doi:jem.20051774 [pii] 10.1084/jem.20051774 (2006).

19 Bossen, C. *et al.* The chromatin remodeler Brg1 activates enhancer repertoires to establish B cell identity and modulate cell growth. *Nat Immunol* **16**, 775-784, doi:10.1038/ni.3170 (2015).

20 Lin, Y. C. *et al.* A global network of transcription factors, involving E2A, EBF1 and Foxo1, that orchestrates B cell fate. *Nat Immunol* **11**, 635-643, doi:10.1038/ni.1891 (2010).

21 Whyte, W. A. *et al.* Master transcription factors and mediator establish super-enhancers at key cell identity genes. *Cell* **153**, 307-319, doi:10.1016/j.cell.2013.03.035 (2013).

22 Smeenk, L. *et al.* Molecular role of the PAX5-ETV6 oncoprotein in promoting B-cell acute lymphoblastic leukemia. *EMBO J* **36**, 718-735, doi:10.15252/embj.201695495 (2017).

23 Mullen, A. C. *et al.* Master transcription factors determine cell-type-specific responses to TGF-beta signaling. *Cell* **147**, 565-576, doi:10.1016/j.cell.2011.08.050 (2011).

24 Lin, Y. C. *et al.* Global changes in the nuclear positioning of genes and intra- and interdomain genomic interactions that orchestrate B cell fate. *Nat Immunol* **13**, 1196-1204, doi:10.1038/ni.2432 (2012).

25 Hill, L. *et al.* Wapl repression by Pax5 promotes V gene recombination by Igh loop extrusion. *Nature* **584**, 142-147, doi:10.1038/s41586-020-2454-y (2020).

26 Ramamoorthy, S. *et al.* EBF1 and Pax5 safeguard leukemic transformation by limiting IL-7 signaling, Myc expression, and folate metabolism. *Genes Dev* **34**, 1503-1519, doi:10.1101/gad.340216.120 (2020).

27 Schwickert, T. A. *et al.* Stage-specific control of early B cell development by the transcription factor Ikaros. *Nat Immunol* **15**, 283-293, doi:10.1038/ni.2828 (2014).

28 Choi, N. M. *et al.* Deep sequencing of the murine IgH repertoire reveals complex regulation of nonrandom V gene rearrangement frequencies. *J Immunol* **191**, 2393-2402, doi:10.4049/jimmunol.1301279 (2013).

29 Verma-Gaur, J. *et al.* Noncoding transcription within the Igh distal V(H) region at PAIR elements affects the 3D structure of the Igh locus in pro-B cells. *Proc Natl Acad Sci U S A* **109**, 17004-17009, doi:10.1073/pnas.1208398109 (2012).
