# Supplementary material for: An Igh distal enhancer modulates antigen receptor diversity by determining locus conformation
Source: Nat Commun. 2023 Mar 3;14:1225. doi: 10.1038/s41467-023-36414-2 (PMC9984487; doi:10.1038/s41467-023-36414-2)
Supplement: Supplementary file 3 — Reporting Summary [file 41467_2023_36414_MOESM3_ESM.pdf]

nature portfolio

Corresponding author(s): A.L. Kenter, NCOMMS-22-33840

Last updated by author(s): 2022/09/05

Reporting Summary

Nature Portfolio wishes to improve the reproducibility of the work that we publish. This form provides structure for consistency and transparency in reporting. For further information on Nature Portfolio policies, see our Editorial Policies and the Editorial Policy Checklist.

Statistics

For all statistical analyses, confirm that the following items are present in the figure legend, table legend, main text, or Methods section.

n/a	Confirmed	
☐	☒	The exact sample size (*n*) for each experimental group/condition, given as a discrete number and unit of measurement
☐	☒	A statement on whether measurements were taken from distinct samples or whether the same sample was measured repeatedly
☐	☒	The statistical test(s) used AND whether they are one- or two-sided<br>*Only common tests should be described solely by name; describe more complex techniques in the Methods section.*
☒	☐	A description of all covariates tested
☒	☐	A description of any assumptions or corrections, such as tests of normality and adjustment for multiple comparisons
☒	☐	A full description of the statistical parameters including central tendency (e.g. means) or other basic estimates (e.g. regression coefficient) AND variation (e.g. standard deviation) or associated estimates of uncertainty (e.g. confidence intervals)
☐	☒	For null hypothesis testing, the test statistic (e.g. *F*, *t*, *r*) with confidence intervals, effect sizes, degrees of freedom and *P* value noted<br>*Give P values as exact values whenever suitable.*
☒	☐	For Bayesian analysis, information on the choice of priors and Markov chain Monte Carlo settings
☒	☐	For hierarchical and complex designs, identification of the appropriate level for tests and full reporting of outcomes
☒	☐	Estimates of effect sizes (e.g. Cohen's *d*, Pearson's *r*), indicating how they were calculated

Our web collection on statistics for biologists contains articles on many of the points above.

Software and code

Policy information about availability of computer code

Data collection	All data analysis tools mentioned in the manuscript have been documented.
Data analysis	GraphPad Prism software, Hi-C was analyzed using Juicer (v1.5;CPU version) and Hi-C heatmaps were generated using Juicebox. 4C interaction matrices were generated from hic files in Juicebox. FACS data was analyzed with FlowJo (v10.8.1) or Summit software; guide RNA sequences were designed using a combination of the http://crispr.mit.edu and BLAST software; DNA FISH data was deconvoluted using Imaris (version 9.0) software. For VDJ-seq unique CDR3s were identified using MiXCR. Hi-C difference maps were assembled by quantile normalization of Hi-C contact frequencies using a null distribution. Source code for the null model chromatin folding by fractal Monte Carlo is available via git repository at https://bitbucket.org/aperezrathke/chr-folder and step by step procedure to generate the null distribution is explained at https://bitbucket.org/aperezrathke/chr-folder under “Null distribution” subsection.

For manuscripts utilizing custom algorithms or software that are central to the research but not yet described in published literature, software must be made available to editors and reviewers. We strongly encourage code deposition in a community repository (e.g. GitHub). See the Nature Portfolio guidelines for submitting code & software for further information.

Data

Policy information about availability of data

All manuscripts must include a data availability statement. This statement should provide the following information, where applicable:

- Accession codes, unique identifiers, or web links for publicly available datasets
- A description of any restrictions on data availability
- For clinical datasets or third party data, please ensure that the statement adheres to our policy

The data sets generated in the course of the current study are available in the GEO repository, accession numbers GSE203484 and GSE201357.

Human research participants

Policy information about studies involving human research participants and Sex and Gender in Research.

Reporting on sex and gender	N/A
Population characteristics	N/A
Recruitment	N/A
Ethics oversight	N/A

Note that full information on the approval of the study protocol must also be provided in the manuscript.

Field-specific reporting

Please select the one below that is the best fit for your research. If you are not sure, read the appropriate sections before making your selection.

☒ Life sciences ☐ Behavioural & social sciences ☐ Ecological, evolutionary & environmental sciences

For a reference copy of the document with all sections, see nature.com/documents/nr-reporting-summary-flat.pdf

Life sciences study design

All studies must disclose on these points even when the disclosure is negative.

Sample size	In all cases at least three independent samples were analyzed and more when needed to attain statistical significance. Total sample size was determined observationally. Pilot studies were undertaken with a least three independent samples each of WT (control) and test samples. The results were assessed and statistically evaluated. If the p value was equal to or less than 0.05 then no additional samples were tested. If the differences between conditions or genotypes approached statistical significance (p equal to or less than 0.05) then we added samples incrementally until statistical power was achieved. This was doable since our sample cohorts rarely exceeded n=10.
Data exclusions	When one technical replicate diverged from a set of three (from one independent sample) then it was excluded.
Replication	All FACS and PCR assays were analyzed in duplicate or triplicate. If one technical replicate diverged from the others in the sample set the analysis was repeated with technical replicates. If the technical replicates failed to reproduce another independent sample was analyzed. All attempts at replication were successful. Occasionally, a technical replicate failed due to technical reasons. In this case the samples in question were re-assessed as compared to the controls and other KOs. If there was still a problem we eliminated that sample.
Randomization	All our comparisons were of controls to genetically altered cell lines or mice that were evaluated using the alternative hypothesis approach. Randomization was not required.
Blinding	As noted above, all our comparisons were focused on controls and genetically altered cell lines or mice and evaluated using the alternative hypothesis approach. Blinding was not required as we were testing the effect of genetic alteration on a function in comparison to the control.

Reporting for specific materials, systems and methods

We require information from authors about some types of materials, experimental systems and methods used in many studies. Here, indicate whether each material, system or method listed is relevant to your study. If you are not sure if a list item applies to your research, read the appropriate section before selecting a response.

Materials & experimental systems

n/a	Involved in the study
	[x] Antibodies
	[x] Eukaryotic cell lines
[x]	Palaeontology and archaeology
	[x] Animals and other organisms
[x]	Clinical data
[x]	Dual use research of concern

Methods

n/a	Involved in the study
[x]	ChIP-seq
	[x] Flow cytometry
[x]	MRI-based neuroimaging

Antibodies

Antibodies used

Antibodies /Reagents Name/Clone Source Identifier
CD19-FITC Rat anti mouse monoclonal/B4 Biolegend Cat#115505
RRID:AB_313640
CD93 PECy7 PE/Cyanine7 anti-mouse CD93/AA4.1 (RUO) Biolegend Cat#136506; RRID_AB_2044012
IgM Fab-AF647 Alexa Fluor® 647 AffiniPure Fab Fragment Goat Anti-Mouse IgM/ Polyclonal Jackson Immuno Research Cat#115-607-020; RRID:AB_2338932
CD2-PE PE anti-mouse CD2 antibody/RM2-5 (RUO) Biolegend Cat#100108; RRID:AB_2073690
CD43-BV421 BV421 Rat anti-mouse CD43/S7 (RUO) BD Biosciences Cat # 562958; RRID:AB_2738069
B220-APC-Cy7 CD45R (B220) monoclonal antibody/RA3-6B2 (APC-eFluor 780) eBioscience Cat # 47-0452-82
RRID:Ab_1518810
CD19-PerCp PerCP anti-mouse CD19 antibody/6D5 (RUO) Biolegend Cat # 115532
RRID:AB_2665409
CD43 (S7)-APC APC rat anti-mouse CD43/S7 (RUO) BD Biosciences Cat # 560663
RRID:AB_1727479
IgM-e450 IgM Monoclonal antibody/ eB121-15F9 (eFluor 450) eBioscience™ Cat # 48-5890-82
RRID:AB_10671539
CD93-PE Cy7 CD93 monoclonal antibody/AA4.1 (PE-Cyanine7) eBioscience™ Cat # 25-5892-81
RRID:AB_469658
IgD-AF700 Alexa Fluor® 700 anti-mouse IgD antibody/11-26c.2a (RUO) Biolegend Cat # 405730
RRID:AB_2563341
CD5-PE PE anti-mouse CD5 antibody/53-7.3 (RUO) Biolegend Cat # 100608
RRID:AB_312737
CD23-biotin Biotin Rat Anti-Mouse CD23
/B3B4 (RUO) BD Biosciences Cat #553137
RRID:AB_394652
CD21-FITC Rat Anti-CD21 / CD35 Monoclonal Antibody/7G6 BD Biosciences Cat #553818
RRID:AB_395070
Streptavidin-APC Streptavidin-allophycocyanin antibody BD Biosciences Cat #554067
RRID:AB_10050396
CD16/32 Fc Block Purified anti-mouse CD16/32 Antibody/93 (RUO) Biolegend Cat# 101302; RRID:AB_312801
CTCF Rabbit Anti-CTCF Polyclonal antibody Millipore
Sigma Cat# 07-729
RRID:AB_441965

Validation

The RRID:Ab identifier linked to the RRID Portal was included for each antibody. The RRID:Ab links to the vendors product sheet and to any published papers that report using the antibody. We relied on the vendors product description for validation.We went further to show specificity by staining with the primary or secondary antibody alone and confirmed no background for the concentrations used in our assays.

Eukaryotic cell lines

Policy information about cell lines and Sex and Gender in Research

Cell line source(s)

The Abelson-MuLV transformed (Abl-t) pro-B cell line, 445.3 (Rag1-/-) on the C57Bl/6 background was kindly provided by Dr. B. Sleckman (University of Alabama at Birmingham).

Authentication

We validated that the Abelson line could be induced to undergo V(D)J recombination following treatment with STI571. We verified that following treatment with STI571 (Gleevac) V(D)J recombination could occur when complemented by a RAG1 expression vector in stable transformants.

Mycoplasma contamination	N/A
Commonly misidentified lines (See ICLAC register)	No commonly misidentified cell lines were used.

Animals and other research organisms

Policy information about studies involving animals; ARRIVE guidelines recommended for reporting animal research, and Sex and Gender in Research

Laboratory animals	C57BL/6 (WT) and Rag deficient mice on the C57BL/6 background were purchased from Jackson Laboratories or maintained in colonies at the University of Illinois College of Medicine, or Scripps Research. NE1-/-, Rag1-/-NE1-/- or Rag2-/-NE1-/- mice all on the C57BL/6 background were constructed and maintained in colonies at the University of Illinois College of Medicine, or Scripps Research. Bone marrow, peritoneal B cells, and splenocytes were harvested from mice 7-12 weeks of age. Mice were evenly balanced for gender.Housing conditions are now specified in the manuscript.
Wild animals	No wild animals were used.
Reporting on sex	We found no discrepancies between animals of different genders.
Field-collected samples	No field collected samples were used in this study.
Ethics oversight	All procedures involving mice were approved by the Institutional Animal Care Committee of the University of Illinois College of Medicine, and the Scripps Research Institute, in accordance with protocols approved by the UIC and Scripps Research Institute Institutional Animal Care and Use Committees.

Note that full information on the approval of the study protocol must also be provided in the manuscript.

Flow Cytometry

Plots

Confirm that:

- [x] The axis labels state the marker and fluorochrome used (e.g. CD4-FITC).
- [x] The axis scales are clearly visible. Include numbers along axes only for bottom left plot of group (a 'group' is an analysis of identical markers).
- [x] All plots are contour plots with outliers or pseudocolor plots.
- [x] A numerical value for number of cells or percentage (with statistics) is provided.

Methodology

Sample preparation	All bone marrow B cell lineage analyses come from CD19+ cells that were expanded in IL7 for 4-5days. Total splenic B cells were analyzed for various subsets based on surface epitope profiles. Peritoneal B cells were isolated and analyzed for B1 B cell subsets.
Instrument	Samples were analyzed on a CyAn ADP (Becton Coulter) or an Attune flow cytometer (Invitrogen).
Software	Summit software (for the CyAn instrument) and FlowJo software (for the Attune instrument.)
Cell population abundance	CD19+ B cells in the spleen represent ~40% of live cells. The starting population leading to B1 B cells in the spleen was CD19+ B220lo CD43+ and represented ~6% of live cells. In the BM, B lineage cells were analyzed using the standard Hardy fraction approach.
Gating strategy	All B cell related gating strategies started with CD19+ cells.

- [x] Tick this box to confirm that a figure exemplifying the gating strategy is provided in the Supplementary Information.
